# Robotic Surgical Training in the Northern Deanery: A trainee-led evaluation in line with GIRFT recommendations

**DOI:** 10.1007/s11701-026-03256-1

**Published:** 2026-03-11

**Authors:** Jada Saunders, Alexander Green, Sreemoyee Ghosh, Mark McKeever, William Fowler, Edward J Nevins, Neena Randhawa

**Affiliations:** 1 Northern Surgical Trainee Research Association (NOSTRA), North East of England, UK; 2 Department of General Surgery, Northumbria Specialist Emergency Care Hospital, Northumbria Healthcare NHS Foundation Trust, Northumbria Way, Cramlington, NE23 6NZ UK; 3 Department of General Surgery, Royal Victoria Infirmary, Newcastle Hospitals NHS Foundation Trust, Queen Victoria Rd, NE1 4LP Newcastle upon Tyne, England, UK; 4 Department of General Surgery, Carlisle Infirmary, North Cumbria Integrated Care NHS Foundation Trust, Newtown Rd, CA2 7HY Carlisle, England, UK; 5 Department of Urology, Sunderland Royal Hospital, South Tyneside and Sunderland NHS Foundation Trust, Kayll Road, Sunderland, SR4 7TP UK

**Keywords:** Robotic, Robotic-assisted, Surgery, Training, Simulation

## Abstract

Robotic-assisted surgery (RAS) has transformed UK surgical practice, with GIRFT emphasising the need for structured and equitable training. This study explores trainee experiences of robotic surgery training within the first UK deanery to adopt a centralised robotic surgery programme. A mixed-methods cross-sectional survey was distributed to surgical trainees in the Northern Deanery, collecting quantitative data on RAS experience, expectations, and training provision. Qualitative data on perceived benefits and barriers, was analysed thematically. There were 60 survey respondents. Support for formal RAS training was near-universal (97%), and most trainees considered RAS relevant to current training (87%), and important for the future of their specialty (90%). However, 80% perceived access to RAS as inequitable across trusts within the region. Most trainees felt competent to observe robotic cases, complete simulation modules, and bedside assist by ST3–4 level, with many reporting readiness for supervised console operating during mid-stage higher training. Mean exposure included 21 ± 21 observed cases, 10.3 ± 12.8 simulation hours, 25 ± 30 bedside-assisted cases, and 4 ± 9 console cases. Perceived impacts of RAS on training were mixed: 21% reported positive effects, 47% negative effects, and 32% neutral. 77% of trainees agreed that access to robotics would influence where they work as a consultant. Thematically, trainees valued “availability of robotic systems”, “consultant engagement and training culture”, “structured training pathways and curriculum”, and “simulation-based training”. Trainees feel competent for early development in simulation, and bedside skills but there remains limited console access locally. There is trainee consensus to necessitate recognition of RAS training formally in the ISCP curriculum.

**Supplementary Information:**

The online version contains supplementary material, including a list of Northern Robotic Collaborators, available at 10.1007/s11701-026-03256-1.

## Introduction

Robotic-assisted surgery (RAS) is increasingly adopted in routine surgical care. The UK Government’s *10 Year Health Plan for England* calls for expansion of robotic technology, with plans to increase surgical robot adoption from 2026 in line with NICE guidelines [1], and NHS England has outlined an ambition for 90% of minimally invasive surgery to be delivered robotically by 2035 [2]. Reflecting this expansion, the *Getting It Right First Time* (GIRFT) *Implementation of RAS in England* report highlights an urgent need for structured, equitable, competency-based RAS training across all surgical specialties [3]. It identifies both a “clear demand for RAS training from residents” and a “significant unmet need for appropriately qualified surgeons” [3]. Despite this, access remains inconsistent and reliant on ad hoc local availability, with a 2020 ASiT survey showing that only 13.5% of trainees had regional access to RAS training and 13.1% within their hospital, despite widespread support for curriculum inclusion [4].

While RAS offers recognised benefits for patients and surgeons, its rapid integration has raised concerns regarding training equity. Without deliberate structuring, robotic exposure may be concentrated among senior trainees or consultants, limiting opportunities for juniors. Despite increasing NHS adoption, the Intercollegiate Surgical Curriculum Programme (ISCP) currently contains no procedure-based assessments (PBAs) for robotic procedures in General Surgery, highlighting misalignment between contemporary practice and national training frameworks [5]. Robotic fellowships are often recommended prior to independent practice but extend training beyond standard pathways [3, 6]. Evidence demonstrates similar early learning curves across junior trainees, robotic novices, and experienced surgeons [7, 8], and well-designed RAS programmes can prepare trainees for independent practice within five years without a fellowship [9]. Delivery of RAS training therefore requires shared responsibility across trainers, trusts, regional programmes, and national bodies, supported by appropriate funding and infrastructure [3, 10].

### Framework of a robotic curriculum

The Royal College of Surgeons of England (RCSEng) guide to RAS describes the stages and standards of:

- Online Training Modules.
- Console or VR based skills simulation (9 h and > 90% scores).
- Observership (including bedside assist > 10 cases).
- Dual Console training.
- Operating surgeon under proctor guidance.
- Sign off for independent robotic practice.

This 2023 RCSEng staged curriculum is evidence based and backed by the 2025 GIRFT recommendations **(**Table 1**)** [10, 11]. This structure has been well adopted by Urological Surgeons in the UK for many years [6]. This curriculum should also be supplemented with mentoring and non-technical skills experience [3].

**Table 1:**
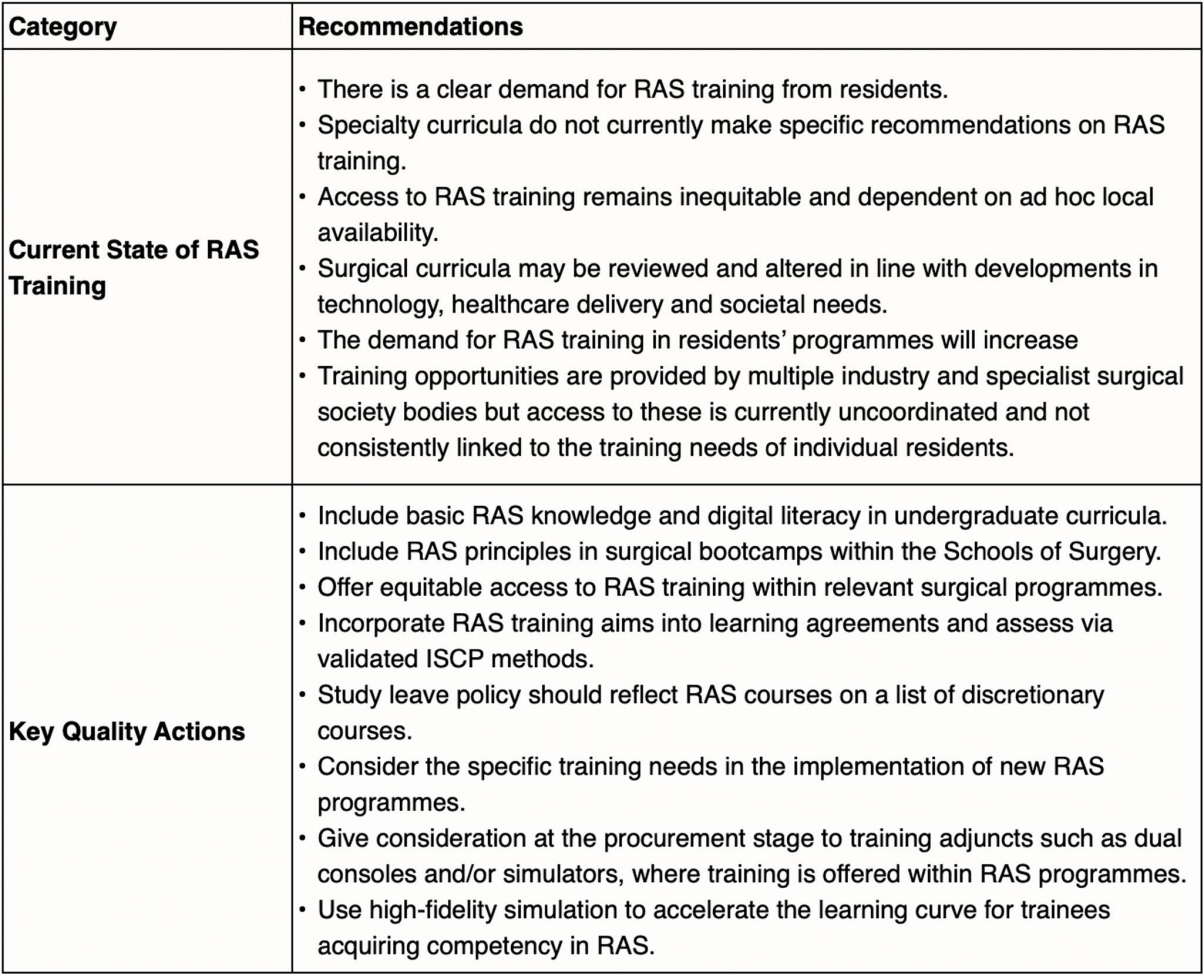
GIRFT RAS training recommendations for residents [3]

### Northern robotics training programme

The Northern Robotics Training Programme, established in 2023, is a phased training pathway delivered through the Northern Surgical Training Centre (NSTC) in collaboration with Intuitive. It was the first UK deanery-wide initiative to provide formalised robotic training to trainees [12]. The programme provides a structured regional framework combining centralised teaching with locally delivered operative exposure across trusts, offering progression through simulation, bedside assisting, and console experience. It is available to surgical trainees at ST5 level and above, with annual deanery training days and competency-based progression typically completed over two to three years.The programme consists of five sequential phases:

- **Phase 0 – Pre-learning**: Completion of online modules to establish foundational knowledge of the robotic platform.
- **Phase 1 – System familiarisation**: An in-person training day introducing platform functions, supported by simulation and procedure-specific educational resources.
- **Phase 2 – Observational and laboratory training**: Observation of robotic procedures with complementary dry and wet lab simulation to develop technical skills.
- **Phase 3 – Intra-operative and advanced skills**: Specialty-specific cadaveric training and supervised intra-operative experience as primary assistant, with a minimum of 10 cases logged under proctor oversight.
- **Phase 4 – Console surgeon training**: Supervised console operating as primary surgeon, with a minimum of 20 cases logged and signed off by a proctor.

#### Training documentation and competency assessment

Progress through the programme is recorded using a dedicated training tracker application, colloquially called the robotic “passport”, which enables documentation of training activities, uploading of evidence, and monitoring of progression through programme phases. This can be used alongside the ISCP portfolio, which documents procedure-specific competencies and graded operative performance, allowing demonstration of both platform-level robotic competence and procedural progression.

#### Specialty-specific integration: Urology

Within Urology, trainees achieving a satisfactory ST5 Annual Review of Competence Progression (ARCP) must complete a Specialist Interest Module (SIM) during their final two training years. As RAS is standard practice for procedures including radical prostatectomy, radical cystectomy, and nephroureterectomy, urological oncology SIMs require a Level 3 sign-off to obtain a Certificate of Completion of Training (CCT). Although this falls short of Level 4 competence required for independent practice, completion of the SIM alongside progression through the Northern Robotics Training Programme supports post-CCT fellowship or mentorship pathways toward independent robotic practice.

#### Programme expansion

Following the success of the Northern Robotics Training Programme, a collaborative initiative, the START programme, has been established between the Newcastle Surgical Training Centre and the Shelford Group. This collaboration commenced in June 2025, with plans for wider rollout of the training programme from December 2025.

### Aim

The primary objective of this study was to evaluate the experiences of surgical trainees regarding RAS training within the Northern Deanery, benchmarked against the GIRFT 2025 RAS training recommendations. The secondary objective was to provide insights and recommendations to support the structured standardisation of RAS training across the region, advocating for a curriculum that is integrated within ISCP and applicable across all training sites.

## Methods

An anonymous online survey was distributed to core and higher surgical trainees (ST1–ST8), including trust-grade doctors, within the Northern Deanery between September and November 2025 via surgical societies, deanery email circulation, and the Northern Surgical Trainee Research Association (NOSTRA) website. Medical students and foundation doctors were excluded. Eligibility was confirmed through General Medical Council registration within the North East, after which responses were anonymised. Data were collected using a secure, password-protected platform accessible only to the first and second authors. Survey questions (Supplemntary Material 2) explored trainee expectations of RAS training and quantified RAS exposure across training stages in relation to GIRFT recommendations, enabling comparison between regionally delivered and trust-based training.The survey was developed using a combination of new and adapted questions. Quantitative (including Likert-scale) and qualitative (free-text) responses were collected. Questions 1 to 6 collect descriptive demographics. Questions from the 2020 ASIT survey [4] were replicated to allow for comparison (questions 7–10 and 12–14). New questions were developed by the authors until there was consensus that questions appropriately assessed training expectations (15–20), experience (21–26), access and documentation (27–28) and qualitative free text questions (29–31). A formal survey pilot was not used.

Descriptive statistics summarised demographics. Continuous variables were analysed using appropriate parametric or non-parametric tests, categorical variables using chi-squared tests, and differences across training grades using ANOVA with post hoc testing where appropriate (IBM SPSS Statistics version 31). Qualitative data were analysed independently by two separate authors, using inductive thematic analysis, with free-text responses independently coded by two authors and themes refined iteratively through discussion, supported by representative quotations. The study followed STROBE reporting guidelines. Participation was voluntary, data were fully anonymised, and formal ethical approval was not required according to the NHS Health Research Authority Research Ethics Tool.

## Results

60 resident doctors completed the survey. All participants completed the single answer survey questions, while 53/60 (88%) completed free text questions. All participants were confirmed to be doctors working within the Northern Deanery. General Surgery accounted for 51 respondents (85%), comprising trust grade SHO (*n* = 1, 2%), CT1/2 (*n* = 8, 13%), trust grade registrar (*n* = 1, 2%), ST3 (*n* = 5, 8%), ST4 (*n* = 15, 25%), ST5 (*n* = 9, 15%), ST6 (*n* = 5, 8%), ST7 (*n* = 5, 8%), and ST8 (*n* = 2, 3%). Urology trainees comprised 9 participants (15%), including trust grade SHO (*n* = 1, 11%), trust grade registrar (*n* = 3, 33%), ST4 (*n* = 1, 11%), ST5 (*n* = 2, 22%), ST6 (*n* = 1, 11%), and ST7 (*n* = 1, 11%). At the time of survey distribution, there were 65 General Surgery and 18 Urology trainees (ST3 and above) in active higher specialty training within the Northern Deanery (total *n* = 83). Of the 60 survey respondents, 46 were higher specialty trainees, representing an estimated response rate of 55% for this subgroup. Responses also included core surgical trainees and trust-grade doctors, for whom a reliable denominator was not available. All respondents had rotated through at least one trust with access to a robotic surgical platform within the preceding three years.

Strong support for RAS was demonstrated (Table 2). Most trainees considered RAS relevant to current training (87%). RAS was also viewed as important for the future of their specialty by 90% of respondents, with near-universal support for a formal RAS training programme (97%). Access to RAS influenced anticipated consultant job choice for 77% of trainees. These positive views aligned with qualitative themes emphasising the value of structured training pathways, early exposure, and access to simulation-based learning. However, most trainees (80%) perceived access to robotic training as inequitable across the regional trusts. Perceptions of RAS impact on current training were mixed: 21% reported positive effects, 47% reported negative effects, and 32% felt neutral. Positive impacts related to early exposure and structured training, with trainees describing “being exposed to robotic lists,” “having access to a simulator,” and valuing “regional training days dedicated to robotic training.” In contrast, negative impacts reflected limited access and competing demands, including “currently working within a trust with no robots,” “trainer learning curves,” and service pressures such as a “busy rota with no time for extra console work” and the “time-consuming nature of the surgery.” Positive and negative impacts were reported similarly across all training grades.

**Fig. 1:**
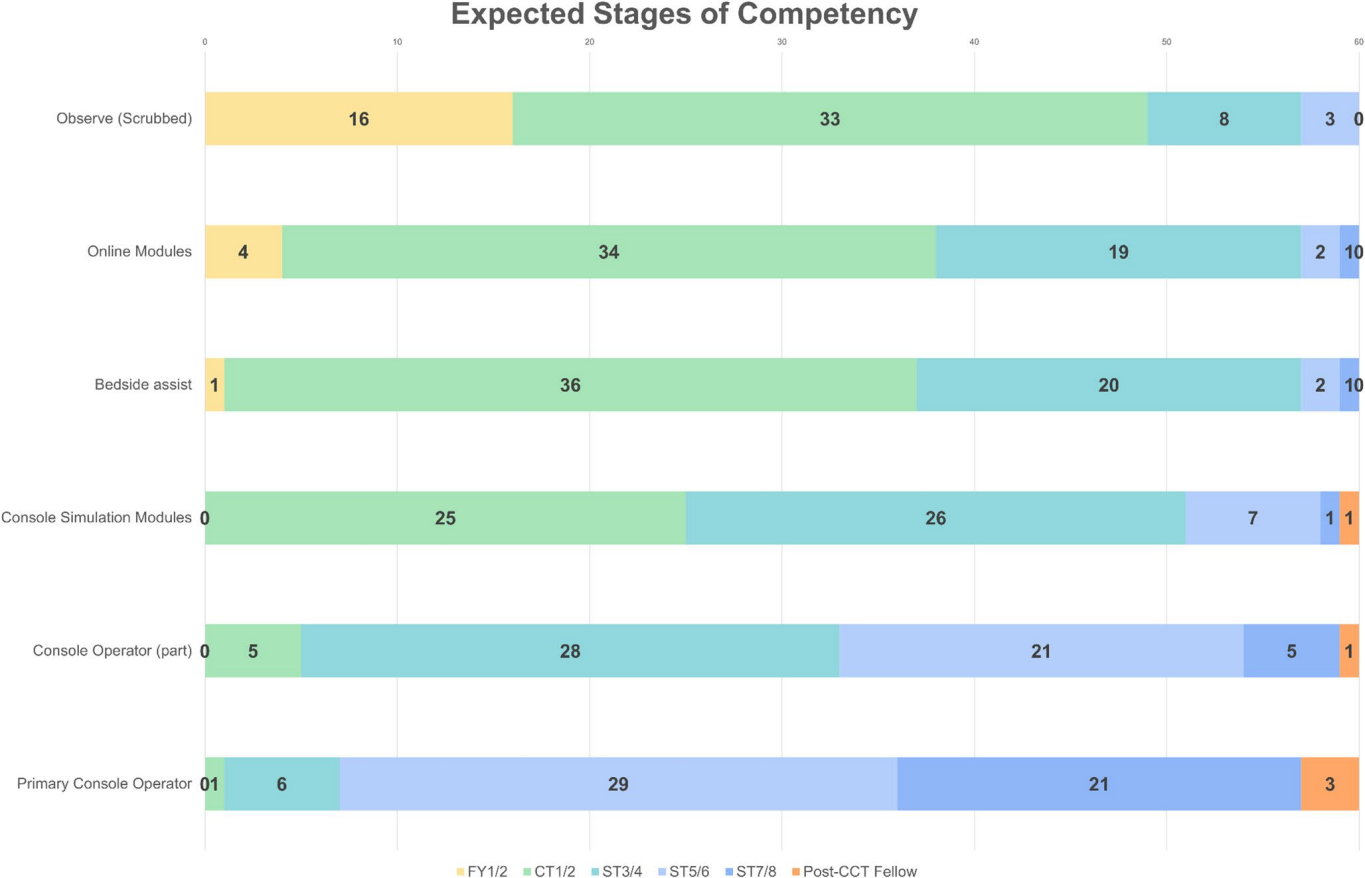
Stacked Bar Chart of Expected Stages of RAS Competency (%)

Preferred training frequency was monthly (32%) or every few months (48%). Funding responsibility was most commonly attributed to training programmes (92%) and training hospitals (52%), with minimal support for trainee-funded models (3%). Expected competency milestones are shown in Fig. 1. Observation (scrubbed) was expected during FY1/2 (27%) or CT1/2 (55%). Online modules (57%) and bedside assisting (60%) were expected during CT1/2. Console-based simulation was anticipated during CT1/2 (42%) or ST3/4 (43%). Performing part of a robotic procedure was expected during ST3/4 (47%) or ST5/6 (35%), with progression to primary console surgeon during ST5/6 (48%) or ST7/8 (35%). Overall, 95% expected to achieve robotic competency before completion of higher surgical training.

**Table 2:** Perspectives and beliefs regarding the role and provision of robotic training

Question	Answers	*n* (%)
Is robotic surgery important for the future of your specialty?	Yes No Unsure	54 (90%) 6 (10%) 0
Is robotic surgery important in current training?	Yes No Unsure	52 (87%) 8 (13%) 0
What impact has robotic surgery had on your training?	Very Negative Slightly Negative Neutral Slightly Positive Very Positive	9 (15%) 19 (32%) 19 (32%) 8 (13%) 5 (8%)
Should RAS be implemented into the formal surgical training programme?	Yes No Unsure	58 (97%) 0 2 (3%)
How often should robotic surgery training be delivered in formal training?	Weekly Monthly Every Few Months Annually	9 (15%) 19 (32%) 29 (48%) 3 (5%)
Who should validate robotic surgery training?	NHS Trusts JCST Royal Colleges of Surgeons Industry	26 (43%) 51 (85%) 44 (73%) 12 (20%)
Who should fund robotic surgery training?	Training Hospitals (NHS Trust) Training Programme (HEE Deanery) Industry Separate Government Funding Trainee	31 (52%) 55 (92%) 21 (35%) 28 (47%) 2 (3%)
You feel that access to robotic training is equitable across training sites in the Deanery.	Strongly Disagree Disagree Neutral Agree Strong Agree	21(38%) 23 (42%) 10 (18%) 1 (2%) 0
Access to robotic surgery would influence my decision on where to work as a consultant.	Strongly Disagree Disagree Neutral Agree Strongly Agree	2 (3%) 2 (3%) 10 (17%) 22 (37%) 24 (40%)

Trainee experience is summarised in Table 3, showing experience by training grade. Exposure was almost exclusively limited to the Intuitive da Vinci platform, with only two trainees also reporting experience with the CMR Surgical Versius system. The mean number of observed robotic cases was 21 (up to 100), with no significant difference by seniority. Most trainees completed online platform modules, but only 35% completed console-based simulation. Simulation exposure varied widely, with 38% reporting none and others reporting up to 40 h; exposure was significantly greater among ST7–8 trainees compared with core trainees (F = 3.339, *p* = 0.011). Bedside assisting was common among higher surgical trainees (mean 27 cases, up to 150). The overall mean (± SD) number of partly or fully performed console cases was 4 (± 9) cases. At ST5/6 level this increased to 6 (± 10) console cases and doubled to a mean of 12 (± 18) console cases in the ST7/8 cohort. One participant reported performing part or all of 40 robotic cases.

**Table 3:** Robotic training experience by trainee level

	Overall Mean ± SD Median (IQR) Range	Training Grade	Mean ± SD	Median (IQR)	*p* value Test Value Test Name
Which robotic platform(s) have you had exposure to in the NHS?	Da Vinci (Intuitive) Versius Surgical System (CMR Surgical) Other	60 (100%) 2 (3%) 0	-		-
How many robotic cases have you observed (scrubbed)?	21 ± 21 10 (25) 0–100	CT/ST1-2 ST3-4 ST5-6 ST7-8 Trust Grade Registrar Trust Grade SHO	12 ± 12 16 ± 17 25 ± 21 28 ± 31 33 ± 22 20 ± 28	10 (10) 10 (17.5) 20 (24.5) 25 (23.75) 40 (40.25) 20 (X)	0.359 F = 1.123 ANOVA
Have you completed the basic required online modules for a robotic platform?	Yes = 43 (72%) No = 17 (28%)	CT/ST1-2 ST3-4 ST5-6 ST7-8 Trust Grade SHO Trust Grade Registrar	38% 86% 71% 86% 50% 50%		0.108 =9.025 Chi-Squared
Have you completed the robotic console simulation modules?	Yes = 21 (35%) No = 39 (65%)	CT/ST1-2 ST3-4 ST5-6 ST7-8 Trust Grade Registrar Trust Grade SHO	13% 29% 47% 63% 25% 0%		0.209 =7.160 Chi-Squared
How many console simulation hours do you have?	10.3 ± 12.8 4 (20) 0–40	CT/ST1-2 ST3-4 ST5-6 ST7-8 Trust Grade Registrar Trust Grade SHO	1.4 ± 2.3† 7.7 ± 9.6 15.7 ± 14.3 21.3 ± 15.8† 2.8 ± 4.9 0 ± 0	0 (4) 2 (16) 15 (21) 22.5 (33.75) 0.5 (7.75) 0 (X)	0.003* F = 4.176 ANOVA
How many robotic cases have you bedside assisted?	25 ± 30 20 (26) 0-150	CT/ST1-2 ST3-4 ST5-6 ST7-8 Trust Grade Registrar Trust Grade SHO	2 ± 5 31 ± 38 26 ± 28 34 ± 26 24 ± 19 10 ± 14	0 (4) 15 (32) 11 (45) 25 (25) 25 (36.25) 10 (X)	0.245 F = 1.383 ANOVA
How many robotic cases have you at least partly performed as a console surgeon (scrubbed)?	4 ± 9 0 (2) 0–40	CT/ST1-2 ST3-4 ST5-6 ST7-8 Trust Grade Registrar Trust Grade SHO	0 ± 0† 0 ± 1† 6 ± 10 12 ± 18† 4 ± 7.5 0 ± 0	0 (0) 0 (0) 0 (4.5) 2.5 (31.25) 0 (11.25) 0 (X)	0.046* F = 2.442 ANOVA

All cases assuming the operation was a routine, index procedure such as a cholecystectomy or right hemicolectomy and in an ideal patient with good supervisor support. F: F-Value, ANOVA: Analysis of Variance, : Pearson Chi-Square Value, †: Statistically significant pairwise comparison with Bonferroni correction, * *p* < 0.05

Provision of RAS training differed between local trusts and deanery-delivered teaching. More trainees accessed RAS training through local trusts than the deanery, including any RAS training (73% vs. 27%), console simulation (55% vs. 28%), bedside assisting (62% vs. 17%), and console operating (25% vs. 13%). Deanery teaching more frequently incorporated dry lab (27% vs. 17%) and wet lab simulation (23% vs. 12%). Elogbook was the most common method to evidence training (52% locally; 35% deanery), with few trainees using DOPS or PBAs on non–robotic-specific ISCP forms. Training courses were more often funded by the deanery (12% vs. 2%), although study leave approval was reported less frequently than for trust-based training.

Thematic analysis identified key facilitators (Table 4) and barriers (Table 5). Positive themes included ‘Availability of Robotic Systems and Lists’, ‘Consultant Engagement and Training Culture’, ‘Structured Training Pathways and Curriculum’, ‘Protected Time and Training Opportunities’, and ‘Simulation-Based Training Opportunities’. Barriers included ‘Limited Robotic Systems and Lists’, ‘Consultant Learning Curve’, ‘Lack of Structured, Standardised Training at Local Level’, ‘Access Disparities and Competition for Bedside, Simulation and Console Training’, and ‘Scheduling Constraints’. Trainee recommendations are summarised in Table 6. Priorities included ‘Curriculum Integration and Standardization’, ‘Access and Early Exposure’, ‘Simulation and Hands-on Training’, and ‘Trainer and Institutional Support’.

**Table 4:** Positive trainee perspectives on robotic surgical training

Theme	Sub-themes	Representative Quotes
**Availability of Robotic Systems and Lists**	Robot presence and quantity	“Working within a trust with two robotic theatres running per day created great opportunities to sit at the second console and assist.” “Having a regular robotic list with trained robotic surgical assistant support.“ “Availability of robots, number of robotic lists, availability of training courses.”
	Robotic case volume	
**Consultant Engagement and Training Culture**	Willingness to teach	“Trainee-focused Consultants.” “Being in a trust where consultants are themselves now confident robotic surgeons.” “Consultants are becoming more comfortable with the platform meaning that dual Consultant operating is less common.” “Consultants willing and keen to teach robotic surgery.”
	Trainer experience and confidence	
	Training culture and attitudes	
**Structured Training Pathways and Curriculum**	Formal programs and courses	“Being in a trust where the trainers are proactive in getting you set up for learning modules on the intuitive site.” “The START programme is sufficient, with extra fellowship if robotics is desired.” “Structured approach to console (starting with bedside training then sim hours and proctoring on the console).”
	Curriculum integration	
	Standardized competencies	
**Protected Time and Training Opportunities**	Dedicated lists and training days	“Regional training days dedicated to robotic training e.g. NTSC sessions.” “Simpler Case selection (Cholecystectomy & hernia) for trainees to begin with.” “Scheduled access for trainees in robotic surgery.” “Surgical SCP to assist so we can get console time.”
	Protected practice time	
**Simulation-Based Training Opportunities**	Access within hours	“Having access to a simulator.” “Self initiative; coming in on my free time to do sim modules.” “NTSC having access to a robot to complete simulations.” “Being exposed to robotic surgery and simulator before getting training number.“
	Dedicated simulation facilities	
	Early simulation integration	

Inductive Thematic Analysis of Positive Perspectives on Robotic Surgical Training: Themes, Subthemes, and Representative Quotes

**Table 5:** Trainee perspectives of barriers to robotic surgical training

Theme	Sub-themes	Representative Quotes
**Limited Robotic Systems and Lists**	Robot presence and quantity	“Currently working within a trust with no robots.” “Low number of robots and robotic lists, high number of trainees seeking access.” “Time consuming nature of the surgery leading to reluctance from theatre staff/anaesthetics.”
	Robotic case volume	
	Service pressures	
**Consultant Learning Curve**	Trainer experience and confidence	“Consultants still training, limited steps given by trainers to trainees, limited training lists.” “Consultants willing and keen to teach robotic surgery.”
	Training culture and attitudes	
**Lack of Structured**,** Standardized Training at Local Level**	Absence of formal structure	“Lack of formal training, steep learning curve.” “Each trust wanting different things/repeating bedside assists.” “High governance requirements in some trusts.” “Requirements to attend local robotic training at each hospital in addition to the regional training program.”
	Redundant requirements across trusts	
**Access Disparities and Competition for Bedside**,** Simulation and Console Training**	Competition for console time	“Competing for console time with other trainees.” “Volume and other more senior trainees, fellows and even consultants wanting to be trained on the robot.” “Surgical care practitioners and scrub nurses being given priority in training in bedside assist roles” “Hospital policies around who can and cannot start training/doing sim on the device.”
	Allied staff involvement	
	Seniority-based access	
	Inconsistent opportunities	
**Scheduling Constraints**	Competing rota commitments	“Busy rota with no time for extra console work.” “Limited access to bedside assistance courses (requirement to be at least ST5).” “Need to complete the simulation but the robot is only free overnight so I have to wait until night shifts and can only do it if I’m not busy. “
	Access within hours	

Inductive Thematic Analysis of Perspectives on Barriers to Robotic Surgical Training: Themes, Subthemes, and Representative Quotes

**Table 6:** Trainee recommendations to improve robotic surgical training

Theme	Sub-themes	Representative Quotes
**Simulation and Hands-on Training**	Simulation access (simulators, modules)	“Formal training days/courses.” “Everyone gets regional access to a training platform for simulation.” “Regular sessions on the training modules; wet lab training.” “Out of work training including doing the modules and cadaveric training.”
	Dedicated training sessions/courses	
	Cadaveric (wet-lab) practice	
**Curriculum Integration & Standardization**	Integrate robotics in curriculum (mandatory training)	“Incorporating robotic surgery into the curriculum to ensure trainees are assigned robotic lists and given opportunities.” “Formalised curriculum, tracked progress across trusts, early involvement in robotic training (online and simulator modules).” “A centralised portfolio from JCST/the deanery detailing competencies and sign offs so you don’t have to ‘start again’ at every trust.” “Access to robotic training passport from st3 to allow early capturing of robotic skills.” “Formal standardized pathway as part of mandatory curriculum”
	Structured pathway with progressive milestones	
	Standardized competencies accepted across all trusts (portfolio/passport)	
**Access and Early Exposure**	Earlier introduction at junior levels (Core training, SHO)	“Start introduction to at least bedside assisting/simulation from core stage.” “Need to implement from an earlier stage – SHOs should be able to do bedside assist with moving on to operating earlier in registrar training.” “Identify robotic ‘hubs’ and allow trainees to travel outside of their primary placement location.” “Scheduled access for trainees in robotic surgery.”
	Wider access across region (more robots or hub travel)	
	Regular allocated opportunities (robotic lists, simulation time)	
**Trainer and Institutional Support**	Consultant upskilling (proctorship)	“Increase proctoring for consultants to get all willing consultants established as robotic surgeons and trainers.” “Encourage consultants to train residents to sit on the console more often.” “Requirements for hospitals to allow regs access to console.” “Must have agreement and engagement from the trusts.” “dedicated person for registering trainees in the trust.”
	Trainer engagement (trainees given console time)	
	Institutional commitment (policies, coordination)	

Inductive Thematic Analysis of Perspectives of Trainee Recommendations to Improve Robotic Surgical Training: Themes, Subthemes, and Representative Quotes

## Discussion

This study provides the first trainee-led evaluation of a structured, deanery-wide RAS training programme in the UK. It benchmarks trainee experience against the 2025 GIRFT recommendations during a period of rapid national expansion of robotic surgical services. Since 2023, the Northern Deanery alongside Intuitive has pioneered a formal regional robotic training pathway, reflecting early and proactive investment in robotic training infrastructure. Evaluating trainee experience within this context provides insight into how structured RAS programmes are experienced in practice and highlights considerations relevant to the wider rollout of robotic training nationally. The findings demonstrate strong trainee engagement with RAS and enthusiasm for a formal curriculum, but also reveal substantial variation in access, training opportunities, and support across local trusts, reflecting the wider national landscape. Almost all trainees recognised the importance of RAS for the future of their specialty (90%) and supported formal inclusion within training programmes (97%), consistent with national data showing increasing trainee demand for robotic training [4]. Notably, 77% reported that access to RAS would influence their choice of consultant post, highlighting the implications of robotic training for workforce planning and recruitment.

When trainee experience was benchmarked against the 2025 GIRFT recommendations, clear patterns emerged. Early-stage milestones, including observation, online learning, and bedside assisting, were generally achieved within the current training landscape. In contrast, progression to later milestones, such as structured simulation, dual-console exposure, and supervised console operating, was less consistently delivered. This indicates that while foundational robotic exposure is established, progression to advanced, competency-based console training remains variable relative to GIRFT aspirations. Despite the presence of a regional robotic training programme, the majority of trainees (80%) perceived access to RAS training as inequitable across trusts within the region. Trainees frequently cited the ‘availability of robotic systems’, ‘access to robotic lists’, and ‘training culture within individual teams’ as key determinants of opportunity. Several described RAS training as dependent on ‘being in the right place’, reflecting inconsistent and poorly standardised local provision. This perception of regional variation is supported by national data, with a 2024 UK-wide survey of 112 hospitals reporting that up to 70% of both trainees and consultants had no access to formal RAS training [13]. While deanery-level robotic training provides an important structured foundation, it is delivered on an annual basis, limiting opportunities for iterative skill development and increasing reliance on trust-level engagement for ongoing progression. Almost half of trainees (48%) felt that RAS training should occur every few months rather than annually (5%), underscoring the need for consistent local integration of regional training structures.

Trainee exposure within this cohort was almost exclusively limited to a single robotic platform. All trainees reported experience with the Intuitive da Vinci system, with only two participants also exposed to the CMR Surgical Versius platform. While platform consistency may support early skill acquisition, it is also likely to intensify competition for console time and constrain training capacity in high-demand settings. Although local hospital trusts provided greater access to simulation, bedside assisting, and console operating than deanery-level teaching (55% vs. 28%, 62% vs. 17%, and 25% vs. 13%, respectively), operative console experience remained modest. The entire cohort reported a mean of four (± 9) robotic cases performed in part as console surgeons. By level, this increased to six (± 10) cases at ST5/6 and then twelve (± 18) cases on average at ST7/8 level. Qualitative data highlighted examples of effective local delivery of robotic training, with some trusts providing trainees with meaningful access to bedside assisting and supervised console exposure within supportive departmental cultures. Consultant engagement and a proactive training ethos were consistently identified as key enablers of trainee progression. However, qualitative data also revealed marked variability in local delivery. In some trusts, trainees reported no access to robotic training, while in others, restrictive governance processes created additional barriers, including mandatory repetition of bedside assisting competencies following trust transfer. Limited availability of RAS-trained consultants, lack of structured opportunities, and competing service demands were commonly cited barriers, aligning with GIRFT’s emphasis on institutional commitment, access to dual consoles, and protected training time.

Simulation was identified as a critical enabler of early RAS skill acquisition, yet access across the region was highly variable. Although some trainees reported up to 40 h of simulation, 65% had not completed the recommended simulation hours, and up to 38% reported no simulation access. Barriers included limited availability and access restricted to evenings or weekends outside clinical working hours. GIRFT strongly recommends high-fidelity simulation, and the RCSEng curriculum mandates defined simulation milestones [3, 5]. Trainees felt that observation, online modules, and bedside assisting should be introduced during core training or early higher specialty training, enabling progression to supervised console work during mid-stage specialty training. In practice, only 35% had completed console simulation, and supervised console operating remained largely confined to senior trainees. This pattern mirrors national concerns that robotic cases may be preferentially retained by consultants or senior fellows, despite evidence that early, structured RAS training is feasible and safe. UK, European, and US studies have demonstrated that junior trainees can safely acquire foundational robotic skills when supported by structured simulation, bedside assisting, and graded console exposure [14–18].

Beyond local training culture, variability in RAS training reflects misalignment between regional training initiatives and national curriculum and assessment frameworks. GIRFT calls for a recognised, transferable curriculum integrated within ISCP to support equitable access, validated assessment, and coordinated programme design. However, robotic surgery remains minimally represented within national training frameworks, with no procedure-based assessments (PBAs) for robotic procedures in General Surgery and only limited representation within Urology, Otolaryngology and Cardiothoracic curricula (5). While the Northern Robotics Training Programme has introduced a regional robotic “passport” to address this gap, formal recognition within ISCP remains limited, restricting the ability to evidence progression and potentially reducing incentives for consistent trainee exposure. The inequity described mirrors national findings, with UK studies highlighting widespread absence of local robotic curricula and calling for national standardisation [13, 19]. Similar concerns are reported internationally, with a Dutch survey demonstrating limited trainee console exposure despite widespread platform availability and strong support for a national RAS curriculum [20].

Finally, expansion of robotic training must be balanced with preservation of foundational operative skill development, particularly during early training. Perceptions of the impact of RAS on surgical training were mixed, with 21% of trainees reporting positive effects and 47% reporting negative effects, reflecting concerns regarding reduced operative autonomy and access to appropriate training opportunities. As robotic surgery expands, operative case-mix may increasingly shift away from open and laparoscopic approaches, reducing exposure to core techniques during early training. These perceptions are evident nationally. An NHS England learner focus group review involving 19 surgical trainees in Portsmouth reported similar concerns. Increasing robotic case volumes, competition from robotic fellows, and significant service pressures were perceived to reduce training opportunities across resident grades, with robotic fellows accessing cases otherwise suitable for higher trainees and a downstream shift of cases from senior to core trainee level [21]. For junior trainees, acquisition of open and laparoscopic skills remains essential for safe practice, including management of complications and scenarios where robotic access is unavailable. It is also important to recognise that not all trainees intend to pursue subspecialties where RAS is central, thus the enthusiasm for robotic training may vary. These findings highlight the need to appropriately sequence robotic exposure so that expansion of robotic services complements, rather than displaces, early operative skill development and aligns with diverse trainee career trajectories.

### Limitations

This self-reported, cross-sectional survey reflects trainee perceptions at a single time point and is subject to recall and selection bias, with dissemination methods potentially favouring trainees interested in robotics or based in centres with established programmes. The survey was not independently validated or piloted. The sample size was modest and weighted toward General Surgery and Urology, and self-reported case numbers and simulation hours may lack precision. Trainer perspectives, local case volumes, and robotic list availability were not assessed, and exposure was almost exclusively limited to the da Vinci platform, restricting platform-agnostic conclusions. As a single-deanery study, these findings reflect local training structure. While similar issues have been reported elsewhere within the NHS, caution is required when extrapolating beyond the study region.

## Conclusion

This study demonstrates strong trainee engagement with robotic-assisted surgery and a clear demand for early, structured, and equitable training across the Northern Deanery. The regional robotic training programme provides a valuable foundation for early exposure and shared standards, although access to simulation and supervised console experience remains inconsistent and dependent on local resources and training culture. These findings align with national and international evidence showing that early robotic curricula are feasible and beneficial but often limited by variable implementation and misalignment with assessment frameworks. Trainees support further refinement of the existing deanery-wide RAS programme, including clearer progression milestones, validated transferable assessment, and more equitable access across trusts in line with GIRFT recommendations. Importantly, expansion of robotic training should be appropriately sequenced to preserve foundational open and laparoscopic skill development while preparing trainees for an increasingly robotic surgical workforce.

## Supplementary Information

Below is the link to the electronic supplementary material.

Supplementary Material 1

Supplementary Material 2

Supplementary Material 3

## Data Availability

The data supporting the findings of this study were generated from an anonymised trainee survey. Due to the sensitive nature of the data and the potential risk of participant identification, the datasets are not publicly available but are available from the corresponding author on reasonable request.
