# Supplementary material for: Robotic Surgical Training in the Northern Deanery: A trainee-led evaluation in line with GIRFT recommendations
Source: J Robot Surg. 2026 Mar 11;20(1):343. doi: 10.1007/s11701-026-03256-1 (PMC12975779; doi:10.1007/s11701-026-03256-1)
Supplement: Supplementary file 1 — Supplementary Material 1 [file 11701_2026_3256_MOESM1_ESM.docx]

# Training Expectations

| **At what stage of training do you think you could be (or could have been) competent to:** | **FY1/2** | **CT1/2** | **ST3/4** | **ST5/6** | **ST7/8** | **Post-CCT Fellow** |
| --- | --- | --- | --- | --- | --- | --- |
|  | **N (%)** | | | | | |
| Observe (scrubbed) robotic surgical procedures | 16 (27%) | 33  (55%) | 8 (13%) | 3 (5%) | 0 | 0 |
| Complete online E-learning modules for robotic surgery platforms? | 4 (7%) | 34 (57%) | 19 (32%) | 2 (3%) | 1 (2%) | 0 |
| Bedside assist* | 1 (2%) | 36 (60%) | 20 (33%) | 2 (3%) | 1 (2%) | 0 |
| Pass robotic console simulation modules | 0 | 25 (42%) | 26 (43%) | 7 (12%) | 1 (2%) | 1 (2%) |
| Perform at least part of a procedure as the console surgeon* | 0 | 5 (8%) | 28 (47%) | 21 (35%) | 5 (8%) | 1 (2%) |
| Be the primary console surgeon (under Procter supervision | 0 | 1 (2%) | 6 (10%) | 29 (48%) | 21 (35%) | 3 (5%) |

*: Assuming the operation was a routine, index procedure such as a cholecystectomy or right hemicolectomy and in an ideal patient with good supervisor support

# Training Provisions

|  |  | **Local Hospital Trusts** | **Regional Deanery** | **Test Value^1^ p Value** |
| --- | --- | --- | --- | --- |
| **In the past 12 months at your placement(s) have you been provided with:** |  | **N (%) n=60** | **N (%)**  **n=54** |  |
| Access to any robotic surgery training? | Yes  No | 44 (73%)  16 (27%) | 15 (25%)  45 (75%) | 0.189  0.664 |
| Access to a robotic surgery console for simulation training? | Yes  No | 33 (55%)  27 (45%) | 17 (28%)  43 (72%) | 7.216 0.007* |
| Teaching for bedside assisting? | Yes  No | 37 (62%)  23 (38%) | 10 (17%) 50 (83%) | 4.210  0.038* |
| Teaching for console operating? | Yes  No | 15 (25%)  45 (75%) | 8 (13%) 52 (87%) | 9.429 0.002* |
| Which of the following simulation methods have been delivered in your trust? | Dry lab  Wet lab  Console sim  None / Unknown | 10 (17%)  7 (12%)  30 (50%)  23 (38%) | 16 (27%)  14 (23%)  20 (33%)  30 (50%) | n/a |
| Which of the following methods have been well used to capture your trust-based robotic surgery training experience?  (Operative or Simulation) | e-Logbook ISCP (DOPS/PBA)  ISCP (Courses) None | 31 (52%)  15 (25%) 4 (7%) 23 (38%) | 21 (35%)  10 (17%) 7 (12%) 33 (55%) | n/a |
| Have external robotic training courses been provided or funded? | Yes  No | 1(2%) 59 (98%) | 7 (12%) 53 (88%) | 9.985 0.002* |
| Has study leave been reliably approved for you to arrange or attend robotic surgery training? | Yes  No | 21 (35%) 39 (65%) | 14 (23%) 46 (77%) | 15.600 <0.001* |

ISCP: intercollegiate surgical curriculum programme, DOPS: Direct Observation of Procedural Skills, PBA: Procedure Based Assessment, ^1^:Pearson Chi-Square Value, *: P<0.05.
