# Supplementary material for: Robotic Surgical Training in the Northern Deanery: A trainee-led evaluation in line with GIRFT recommendations
Source: J Robot Surg. 2026 Mar 11;20(1):343. doi: 10.1007/s11701-026-03256-1 (PMC12975779; doi:10.1007/s11701-026-03256-1)
Supplement: Supplementary file 2 — Supplementary Material 2 [file 11701_2026_3256_MOESM2_ESM.docx]

# Robotic Surgical Training Survey – Full Questionnaire

## Consent

1. Do you consent to participating in this survey?

- - Yes
- - No

1. Please provide your GMC number to verify you are a doctor working in the Northern Deanery (responses anonymous).

- - Free text

1. If you wish to be involved in future work or co-authorship, please provide your email address.

- - Free text

## Demographics

1. What is your current training level?

- - FY1/2
- - CT1/2
- - ST3
- - ST4
- - ST5
- - ST6
- - ST7
- - ST8
- - Post-CCT Fellow
- - Trust Grade SHO
- - Trust Grade Registrar

1. What is your surgical specialty?

- - General Surgery
- - Urology
- - Obstetrics & Gynaecology
- - Trauma & Orthopaedics
- - ENT
- - Vascular Surgery
- - Plastic Surgery
- - Cardiothoracic Surgery
- - Neurosurgery
- - Other

1. Please select your last trust(s) within the Northern Deanery where you have worked in the last 3 years.

- - County Durham & Darlington
- - Gateshead
- - Newcastle upon Tyne
- - North Tees & Hartlepool
- - Northumbria
- - South Tees
- - South Tyneside & Sunderland
- - North Cumbria

## Attitudes to Robotic Surgery

1. Is robotic surgery important for the future of your specialty?

- - Yes
- - No

1. Is robotic surgery important in your current training?

- - Yes
- - No

1. What impact has robotic surgery had on your training?

- - Very positive
- - Slightly positive
- - Neutral
- - Slightly negative
- - Very negative

1. Should robotic surgery be implemented into the formal surgical training programme?

- - Yes
- - No
- - Unsure

1. Access to robotic surgery would influence my decision on where to work as a consultant.

- - Strongly agree
- - Agree
- - Neutral
- - Disagree
- - Strongly disagree

## Training Structure & Funding

1. How often should robotic surgery training be delivered in formal training?

- - Annually
- - Every few months
- - Monthly
- - Weekly
- - Should not be introduced

1. Who should validate robotic surgery training? (Select all that apply)

- - NHS Trusts
- - JCST
- - Royal Colleges of Surgeons
- - Industry

1. Who should fund robotic surgery training? (Select all that apply)

- - Training hospital
- - Training programme
- - Industry
- - Government
- - Trainee/Surgeon

## Training Expectations

1. At what stage should trainees be competent to observe robotic surgery (scrubbed)?

- - FY1/2
- - CT1/2
- - ST3/4
- - ST5/6
- - ST7/8
- - Post-CCT Fellow

1. At what stage should trainees complete robotic e-learning modules?

- - FY1/2
- - CT1/2
- - ST3/4
- - ST5/6
- - ST7/8
- - Post-CCT Fellow

1. At what stage should trainees be competent at bedside assisting?

- - FY1/2
- - CT1/2
- - ST3/4
- - ST5/6
- - ST7/8
- - Post-CCT Fellow

1. At what stage should trainees pass console simulation modules?

- - FY1/2
- - CT1/2
- - ST3/4
- - ST5/6
- - ST7/8
- - Post-CCT Fellow

1. At what stage should trainees perform part of a procedure as console surgeon?

- - FY1/2
- - CT1/2
- - ST3/4
- - ST5/6
- - ST7/8
- - Post-CCT Fellow

1. At what stage should trainees act as primary console surgeon (under supervision)?

- - FY1/2
- - CT1/2
- - ST3/4
- - ST5/6
- - ST7/8
- - Post-CCT Fellow

## Experience

1. Which robotic platforms have you been exposed to? (Select all that apply)

- - Intuitive da Vinci
- - CMR Versius
- - Hugo RAS
- - Senhance
- - Stryker Mako
- - ROSA
- - VELYS
- - Other

1. Have you completed required online modules?

- - Yes
- - No

1. Have you completed console simulation modules?

- - Yes
- - No

1. How many robotic cases have you observed?

- - Numeric entry

1. How many robotic cases have you bedside assisted?

- - Numeric entry

1. How many robotic cases have you performed part or all of as console surgeon?

- - Numeric entry

## Access & Documentation

1. Access to robotic training is equitable across training sites in the Deanery.

- - Strongly agree
- - Agree
- - Neutral
- - Disagree
- - Strongly disagree

1. How is your robotic training recorded? (Select all that apply)

- - E-logbook
- - ISCP PBAs
- - ISCP DOPS
- - Courses
- - None

## Free Text Responses

1. What has most improved your access to robotic training? (Top 3 factors)

- - Free text

1. What most limits your access to robotic training? (Top 3 barriers)

- - Free text

1. What would you recommend to improve robotic training across the Deanery?

- - Free text
