# Supplementary material for: Robotic Surgical Training in the Northern Deanery: A trainee-led evaluation in line with GIRFT recommendations
Source: J Robot Surg. 2026 Mar 11;20(1):343. doi: 10.1007/s11701-026-03256-1 (PMC12975779; doi:10.1007/s11701-026-03256-1)
Supplement: Supplementary file 3 — Supplementary Material 3 [file 11701_2026_3256_MOESM3_ESM.docx]

## **Collaborative Authors**

### **Darlington Memorial Hospital** Prashant Girijavallabhan Nair, MBBS, MRCS (Eng), PGCert MedEd

### **James Cook University Hospital**

Mohammed Arifuzaman, MBBS, MSc Surg, MRCS (Eng), FMAS, DMAS
Hira Bakhtiar Khan, MBBS, MRCS
Zeeshan Kareem, MBBS, MS, DrNB (Urol), MRCS
Jason Trevis, MRCS

### **Newcastle upon Tyne Hospitals NHS Foundation Trust**

Elizabeth Jones, BMBS
Ahmad O. Khalifa, MBBCh, MSc, MD, FEBU, FRCS (Urol)
Georgios Kourounis, MBBS, MRCS, MSc
Dina Saleh, MBBCh, MRCS

**North Cumbria Integrated Care NHS Foundation Trust**

Shivani Baskar Kuttuva, MBBS, MRCS
Claire Crewe, MBBS, MRCS, MMedEd

### **North East Deanery**

### Fatema Almadhoob, MB BCh, LRCSI, MRCS (Ed) Alex Millward, MBChB, MRCS

### **North Tees and Hartlepool NHS Foundation Trust**

Bertram Marks, MBBS, MRCS, MRes, PGCert (Ed)
Chirag Rao, MBBS, BSc, MRCS (Ed), PGCert MedEd
Ali Ahmed Wuheb, MBBS, MRCS (Ed), MSc, PGDip ClinEd

### **Northumbria Healthcare NHS Foundation Trust**

Mustafa Alqasem, MBChB, MRCS
Gowri Madhusudanan Pillai, MBBS, MS, DNB, MRCS
Jitendra Singh, MBBS, MRCS
Holly Wright, MBBS, MRCS

### **South Tyneside and Sunderland NHS Foundation Trust**

Ahmed Ammar, MD, MRCS
Gowtham Venkatesan, MBBS, DNB, MCh, MRCS
